# Correspondence

**Published:** 1900-04

**Authors:** Rufus B. Hall


*Editor of Journal-Record of Medicine:*

My attention has been very recently called to an article in the January number of THE ATLANTA JOURNAL-RECORD OF MEDICINE. It is a report of a case of an operation for intraligamentous cyst by Dr. Crofford, of Memphis, in which the technic of the operation is described as suggested by myself. The technic used in the description of this operation is not that proposed by me. The very point he criticizes, where he says I do not ligate the uterine artery on the tumor side, is where he is mistaken.

I send in this letter a description of the operation as presented to the Southern Surgical and Gynecological Association in 1897, to verify my statement, also a recent report of a retroperitoneal tumor where the same technic was followed. I always ligate both uterine arteries and both ovarian arteries before commencing the enucleation of the tumor. This is the only way to make a bloodless operation, and by doing that it is a bloodless operation as near as any operation can be. Sometimes, for convenience sake, I clamp the uterine artery on the tumor side temporarily. This controls the hemorrhage until the tumor can be removed. Then this artery can be ligated more convently. I hope that you will make the necessary correction.

Very sincerely yours,

RUFUS B. HALL.

---

### PAINLESS INJECTIONS OF MERCURIALS.

Bazin states that if pure guaiacol be incorporated with the oil used as a medium for intramuscular injections of mercurials the process is rendered practically painless. He gives this formula, the guaiacol being employed in the proportion of three per cent.:

| | | |
|---|---|---|
| ℞ | Hydrarg. iod. rubr........................... | 8 grains. |
| | Guaiacol pur.................................... | 45 minims. |
| | Ol. olivæ ster .................................. | 3 ounces. |

M. Sig. For hypodermic use.

The injections are practiced daily or every other day, 30 minims representing approximately gr. 1-6 of the biniodid. The needle is inserted perpendicularly to the skin, pushed in its full length, and the liquid injected very slowly, the buttocks being chosen preferably for the operation.—*Medical News.*

